# Primary care quality indicators for children: measuring quality in UK general practice

**DOI:** 10.3399/bjgp14X682813

**Published:** 2014-12-01

**Authors:** Peter J Gill, Braden O’Neill, Peter Rose, David Mant, Anthony Harnden

**Affiliations:** The Hospital for Sick Children, Department of Paediatrics, University of Toronto, Toronto, Ontario, Canada.; Cumming School of Medicine, University of Calgary, Calgary, Alberta, Canada.; Nuffield Department of Primary Care Health Sciences, University of Oxford, Oxford, UK.; Nuffield Department of Primary Care Health Sciences, University of Oxford, Oxford, UK.; Nuffield Department of Primary Care Health Sciences, University of Oxford, Oxford, UK.

**Keywords:** clinical guidelines, paediatrics, primary health care, quality indicator

## Abstract

**Background:**

Child health care is an important part of the UK general practice workload; in 2009 children aged <15 years accounted for 10.9% of consultations. However, only 1.2% of the UK’s Quality and Outcomes Framework pay-for-performance incentive points relate specifically to children.

**Aim:**

To improve the quality of care provided for children and adolescents by defining a set of quality indicators that reflect evidence-based national guidelines and are feasible to audit using routine computerised clinical records.

**Design and setting:**

Multi-step consensus methodology in UK general practice.

**Method:**

Four-step development process: selection of priority issues (applying nominal group methodology), systematic review of National Institute for Health and Care Excellence (NICE) and Scottish Intercollegiate Guidelines Network (SIGN) clinical guidelines, translation of guideline recommendations into quality indicators, and assessment of their validity and implementation feasibility (applying consensus methodology used in selecting QOF indicators).

**Results:**

Of the 296 national guidelines published, 48 were potentially relevant to children in primary care, but only 123 of 1863 recommendations (6.6%) met selection criteria for translation into 56 potential quality indicators. A further 13 potential indicators were articulated after review of existing quality indicators and standards. Assessment of the validity and feasibility of implementation of these 69 candidate indicators by a clinical expert group identified 35 with median scores 8 on a 9-point Likert scale. However, only seven of the 35 achieved a GRADE rating >1 (were based on more than expert opinion).

**Conclusion:**

Producing valid primary care quality indicators for children is feasible but difficult. These indicators require piloting before wide adoption but have the potential to raise the standard of primary care for all children.

## BACKGROUND

Child health care is an important part of the UK general practice workload; in 2009 children aged <15 years accounted for 10.9% of consultations with GPs.^1^ However, the UK Quality and Outcomes Framework (QOF), the financial incentives scheme to reward high-quality clinical practice, largely excludes any assessment of care quality for children despite repeated calls for its inclusion.^2^,^3^ In an analysis of the effect of this pay-for-performance initiative on three areas of clinical care between 2003 and 2007, the quality of aspects of care not incentivised by the QOF had declined in two areas and had improved more slowly in the third.^4^ Therefore, omission from the QOF probably has a negative impact on care quality. Although the proportion of funding linked to the QOF is diminishing,^5^ the importance of indicators on which to base audit and clinical governance has not diminished. The consequences of low-quality care of children are reflected in unplanned hospital admissions^6^ and preventable deaths.^7^

In the UK, the National Institute for Health and Care Excellence (NICE) has developed an electronic library of quality standards including a number that are child specific, focused on topics such as cancer and urinary tract infections.^8^ Quality indicators, measurable aspects of care against which quality standards can be set and audited, have been developed for some aspects of primary care in the US, Canada, and the Netherlands.^9^–^13^ A group in the UK has proposed quality indicators for the primary care of child and adolescent mental health,^14^ and indicators potentially relevant to the primary care of children can be found for a number of other disease-specific areas (such as diabetes).^15^ However, there are obvious gaps. This current research attempts to develop a comprehensive set of quality indicators for the primary care of children and adolescents, which include all aspects of paediatric care and reflect existing evidence-based national guidelines.

## METHOD

A multi-step approach was adopted to develop a comprehensive set of quality indicators for the primary care of children and adolescents aged <18 years. The four steps in the development process are summarised in Box 1.

Box 1. **Summary of method for developing quality indicators**

**Table:** 

**Step 1: Selection of priority areas for quality indicator development**	The clinical areas for which priority quality indicators are most needed were identified by an expert review panel using the nominal group technique; the expert panel was informed by evidence obtained by qualitative interviews with practising primary care clinicians, evidence from a systematic review of effective clinical interventions, and an analysis of unplanned hospital admissions for primary care-sensitive conditions.
**Step 2: Identification of relevant recommendations from national guidelines**	All national guidelines for England (NICE) and Scotland (SIGN) were reviewed to select those potentially relevant to children and dealing with issues in the prioritised clinical areas. All recommendations were extracted but were selected for further development only if they: 1) made a precise statement about what constitutes high-quality care; 2) defined a standard against which care quality could be measured; 3) were clearly defined; 4) were measurable; and 5) were attributable to actions in primary care.
**Step 3: Translation of selected guideline recommendations into quality indicators**	To inform the drafting process, exemplar quality indicators were identified from three specific sources: 1) the Agency for Healthcare Research and Quality (AHRQ) National Quality Measures Clearinghouse website; 2) the Royal College of General Practitioners (RCGP) Training Standards and other previously proposed quality indicators for UK general practice; 3) paediatric indicators previously developed by RAND. If the above sources contributed no useful exemplars, PubMed was searched for newly published indicators.
**Step 4: Final assessment of quality indicators for feasibility and reliability**	All the quality indicators were further assessed by a second expert review panel (with some overlap in membership with the panel that undertook Step 1) using the RAND appropriateness method.

The areas and aspects of care of highest priority for quality indicator development were identified by an expert panel of clinicians using the nominal group technique,^16^ a structured process that has been used by others in the development of measures of care quality.^11^ The membership of the group and exact method applied have been described in detail elsewhere.^17^ In brief, it included UK GPs with substantial clinical experience and an interest in child health; they had a range of expertise from commissioning, child protection, medical education and training, academic general practice, to NICE guideline development.

**How this fits in**

Child health care is an important part of general practice workload in the UK, but is very poorly represented in the financially incentivised Quality and Outcomes Framework. Although NICE has developed a small number of relevant quality standards, primary care quality indicators have been developed for specific conditions, and indicator sets for paediatric ambulatory care have been developed in North America and Holland, there remains an important gap. This current research has developed a comprehensive set of quality indicators relevant to the care of children and adolescents. The indicators could be feasibly implemented in the UK by audit of computerised general practice records.

All published NICE and SIGN guidelines were systematically searched to identify recommendations relevant to children and adolescents. The assessment of these recommendations (against the five criteria listed in Box 1) to evaluate their potential to be quality indicators employed a modified version of the iterated consensus rating procedure (which is recommended as the optimal process to identify recommendations with quality indicator development potential).^18^,^19^

The final clinical assessment of feasibility and validity was based on a method devised by the RAND organisation (the most widely used methodology to generate professional consensus on quality indicators).^20^,^21^ The panel (*n* = 13, 10 at final meeting) was of similar composition to that which participated in the nominal group technique rating, with some overlap in membership. It was provided with the GRADE rating for each indicator and referenced source material.^22^ Panel members were required to rate the validity and feasibility of the proposed indicators on a 9-point Likert scale (1 = strongly disagree, 9 = strongly agree).

## RESULTS

### Clinical areas

**Figure 1.**
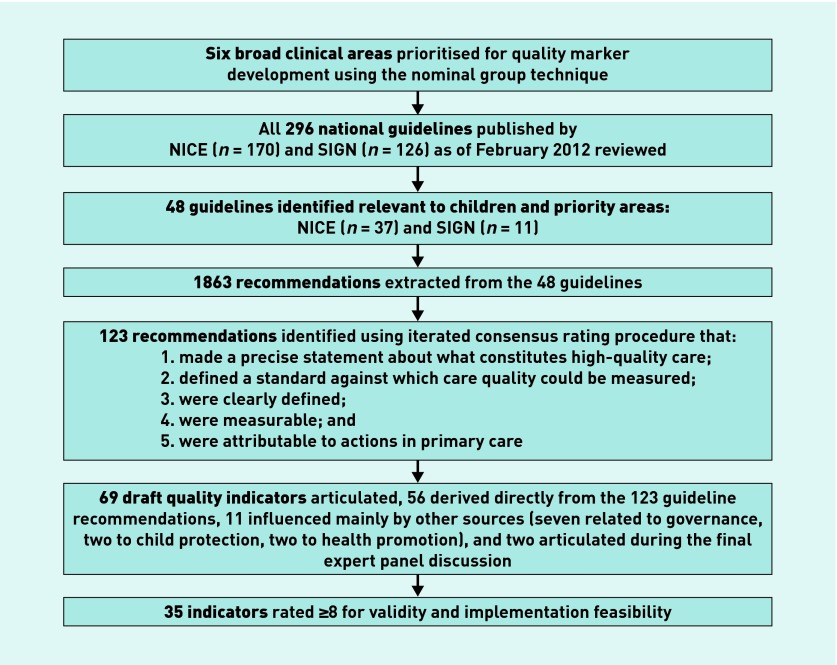
***Outcome of development process.***

Figure 1 summarises the outcome of the development process. The nominal group discussion identified six broad clinical areas in which development of quality was agreed to be important. Three focused on clinical management of illness (early recognition of serious illness, management of common conditions, and mental health) and three on more generic aspects of child care (child protection, health promotion, and governance). The governance issues ranged from illness specific (for example, unnecessary admission of children to hospital with acute asthma) to standards of professional development (for example, review of paediatric-specific skills in annual appraisal).

### National guideline review

Review of the 296 national guidelines identified 48 (37 NICE and 11 SIGN) relevant to children or adolescents, containing 1863 potentially relevant recommendations. However, only 123 (6.6%) met all five selection criteria and these were taken forward for translation into quality indicators. Most of these recommendations were about clinical management of illness (33 early recognition of serious illness; 51 management of common conditions; 28 mental health). Only three recommendations selected were about child protection, eight about health promotion, and none about governance.

### Articulation and assessment of indicators

The 123 recommendations were translated into 56 quality indicators; this reduction mainly reflected overlap (that is, more than one recommendation could be addressed by a single indicator). However, the process of identifying exemplar indicators from other sources not only facilitated drafting but also identified indicators for the generic aspects of clinical care not covered by guideline recommendations. A further 11 indicators were added through this mechanism (seven relating to governance, two each relating to health promotion and child protection) and two articulated by panel members during the final assessment phase. A total of 69 potential quality indicators were therefore included in the final assessment of feasibility and validity, of which 35 achieved a median score of ≥8. Of these 35 indicators, 28 were rated as GRADE 1 (expert consensus) while four were rated 2 (low level of evidence), one was rated 3 (moderate level of evidence) and two were rated 4 (high level of evidence).

### Quality indicators for routine care

Box 2 shows the indicators relating to the quality of routine clinical care. Most cover common conditions seen mainly in pre-school children including acute infection, acute diarrhoea, and allergic reactions. Four indicators relate to more specific age groups: neonatal jaundice, infant colic, and adolescent self-harm. Of the three indicators that are not focused on specific conditions, two are process measures (professional development and the availability of equipment) but one is a health service outcome measure: the number of accident and emergency attendances by children registered with the practice. The panel recognised that this would need to be standardised for other factors such as the age profile of the child population and the practice deprivation index as both will influence accident and emergency attendance rates.

Box 2. **Indicators of the quality of routine clinical care**

**Table:** 

**Acute infections**	Antibiotic prescriptions in children should be accompanied by a clearly documented rationale for this decision
**Acute diarrhoea**	Children ≤5 years with gastroenteritis should have hydration status clearly documented
**Allergic reactions**	Children with an acute allergic reaction to a food substance should be referred for appropriate investigations
**Enuresis**	Children with nocturnal enuresis should have a clearly recorded assessment that differentiates between primary and secondary enuresis Children newly presenting with secondary enuresis should have clearly documented evidence of glucose assessment
**Infant colic**	Infants with colic should not be prescribed dicycloverine (dicyclomine)
**Neonatal jaundice**	Neonates ≥37 weeks (gestational age) with jaundice lasting ≥14 days or neonates <37 weeks (gestational age) with jaundice lasting ≥21 days who present to the GP should have clearly documented evidence of conjugated bilirubin measurement
**Otitis media with effusion**	Children ≥3 years with persistent bilateral otitis media with effusion or any age with speech and language, developmental, or behavioural problems should be referred for hearing assessment
**Self-harm**	Children who self-harm should have a clearly documented assessment and management plan
**Other**	Practices should have access to appropriate growth charts including body mass index (BMI) measurement in children GPs should document written reflection of their paediatric continuing professional development (CPD) activities undertaken within each 5-year revalidation cycle Accident and emergency attendances for children in previous 12-month period

### Quality indicators for chronic illness

Box 3 shows the indicators relating to the quality of recognition and care of chronic illness. Not surprisingly, four are about asthma (the most common chronic disease managed in primary care). The other specific chronic conditions covered were eczema, coeliac disease, diabetes, mental health, epilepsy, hepatitis B risk, and attention deficit hyperactivity disorder (ADHD). The one generic indicator is about long-term prescribing.

Box 3. **Indicators of the quality of recognition and care of chronic illness**

**Table:** 

**ADHD**	Stimulant medication for the treatment of ADHD should not be initiated by GPs
**Asthma**	Children with asthma aged ≤5 years should have a clearly documented basis for diagnosis Children with asthma should be prescribed a spacer Children with asthma should have an annual review with documented height Children and young people admitted or seen in secondary care for an asthma exacerbation should be assessed within 30 days in primary care
**Coeliac disease**	Children with chronic or intermittent diarrhoea and/or faltering growth should be investigated with serological testing for coeliac disease
**Diabetes**	Children newly presenting with polydipsia, polyuria, and/or weight loss should have clearly documented evidence of glucose assessment Children with Type 1 diabetes aged ≥6 months should have documented evidence of being offered annual influenza immunisation
**Eczema**	Children with atopic eczema should be prescribed emollients Percentage of children who have a repeat prescription of moderate/very potent topical steroids Children with atopic eczema with suspected eczema herperticum should be referred urgently for further assessment
**Epilepsy**	Children with a first non-febrile seizure should have clearly documented evidence of referral to secondary care for further assessment
**Hepatitis B risk**	Children eligible for targeted hepatitis B immunisation should have a complete and up-to-date immunisation record
**Long-term prescribing**	Children on long-term prescriptions should have an annual review in primary care Children taking methylphenidate, atomoxetine, or dexamfetamine should have clearly documented monitoring
**Mental health**	Antidepressant medications should not be initiated by GPs for children and young people with depression

### Quality indicators for child protection and developmental assessment

Box 4 lists the indicators selected for child protection and developmental assessment. One indicator, the taking of appropriate urgent action on new-onset fixed squint, is a developmental issue but could equally be seen as an important indicator of high-quality routine care. The other indicators focus on safeguarding; two on professional development and training, and three on recognition of abuse and neglect (although regression in language or motor skills can sometimes have causes other than abuse or neglect).

Box 4. **Indicators of the quality of child protection and developmental assessment**

**Table:** 

**Developmental assessment**	Children ≥3 years with regression in language or any age with regression in motor skills should be referred for further assessment Children with a new-onset fixed squint should be assessed and referred urgently when appropriate
**Neglect or abuse**	Children about whom a practitioner suspects neglect or abuse should have evidence that a clear and recorded course of action was taken ‘Looked-after’ children and young people should be clearly identified in the GP’s summary record ‘Looked-after’ children and young people should have an annual review and an updated personal health record
**Staff professional development**	Relevant staff should know the practice lead and the contact details for the named/designated professionals for safeguarding children All relevant staff must have received child protection/safeguarding of children training in line with local policy

## DISCUSSION

### Summary

Producing quality indicators is difficult. It took 2 years to produce 35 indicators that have high levels of clinical support and reflect national guidelines. These indicators were developed by a multi-step method very similar to that used in formulating the QOF. They cover a range of clinical issues agreed to be of importance to high-quality primary care for children and adolescents. However, there are many important aspects of care that are not covered and most indicators are supported only by low-level evidence (mainly expert opinion). They need to be piloted before large scale implementation to check for unforeseen difficulties in use and adverse consequences but they do fill an important gap. Lack of a set of quality indicators is no longer an excuse for the omission of markers of paediatric care from the QOF.

### Strengths and limitations

The main strength of the research undertaken was that it followed a well-defined multi-step process that has been developed and refined by others. As stated in the methods, the nominal group technique was used by Guttmann *et al*, in defining important clinical areas in which to develop quality indicators for emergency paediatric care in Canada.^11^ The RAND appropriateness method, developed in North America, is now a core feature of the process of development of quality indicators for the UK QOF.^21^,^22^ The initial assessment of the most important areas in which to develop quality indicators was informed by research (a qualitative survey of service GPs^23^ and an analysis of emergency hospital admissions).^6^ Every attempt was made to ensure the indicators were evidence based (by basing the initial drafting on national clinical guidelines prepared by NICE).

The main weakness of the process was that the only stakeholders consulted were GPs. This approach was taken because the initial implementation concern was professional acceptability. In the UK, paediatric care is provided by the whole primary care team, with health visitors in particular playing an important role in promoting child health, safeguarding children, and supporting families of children aged <5 years. If the process was repeated it would involve other primary care clinicians (for example nurses or health visitors) in the expert panels and consultation with other stakeholders (for example, parents, hospital and community paediatricians, or health service commissioners). Limiting the process to clinicians with a professional and financial interest in the outcome has the obvious potential to influence both the areas chosen as important for development and the assessment of validity and feasibility.

The study departed from a strict application of the consensus method by including indicators in the final set based on their median rating for validity and implementation even if complete consensus was not achieved. The authors believe that seeking complete consensus risks a ‘lowest common denominator’ effect, with the exclusion of otherwise highly-rated indicators because they were opposed by one or two individuals with a strong contrary opinion. For example, ‘children with atopic eczema should be prescribed emollients’ achieved median ratings of 8 and 9 but would have been rejected because one panel member gave a 2 rating for both feasibility and validity.

### Comparison with existing literature

Most of the previously developed paediatric quality indicators focus on care of acute illness (for example, diabetic ketoacidosis, status asthmaticus, anaphylaxis, status epilepticus, severe head injury, and sepsis).^24^ The quality indicators used to assess the care of children in the US Medicaid programme looked at primary care but focused on vaccination, access, and procedures.^25^ The set of paediatric quality indicators developed by Giesen in the Netherlands also focused on primary care and used a very similar approach of developing indicators based on national guidelines but focused only on prescribing and referral out-of-hours.^12^ The most inclusive set of primary care quality indicators for children and adolescents was developed in the US by Mangione-Smith *et al* in 2007 using the RAND methodology, but it involves 175 indicators (covering management of acne, ADHD, allergic rhinitis, asthma, depression, diarrhoea, fever, urinary tract infection, and sexually transmitted Infections as well as prevention and care of well children) and is more useful for one-off surveys of care quality rather than routine monitoring and quality assurance.^10^

There is some overlap between our work and the quality standards that NICE are starting to publish, some of which apply to children.^8^ For example, the NICE standard for primary care of epilepsy is that ‘Children and young people presenting with a suspected seizure are seen by a specialist in the diagnosis and management of the epilepsies within 2 weeks of presentation.’ This study’s comparative indicator is formulated more precisely in terms of the children who needed to be referred but it is less precise on timing: ‘Children with a first non-febrile seizure should have clearly documented evidence of referral to secondary care for further assessment.’^26^

The varying language in which our indicators are expressed highlights the overlap between the concepts of a quality indicator and quality standard. Some are expressed as true indicators without any predefined standard (for example, accident and emergency attendances for children in previous 12-month period) while others imply a zero-tolerance standard (for example, infants with colic should not be prescribed dicyclomine). In order to apply the indicators in clinical practice it is necessary to set a standard (that is, the level which must be achieved) but it should be noted that QOF indicators seldom set a zero-tolerance standard: full points are awarded for less than 100% compliance.

### Implications for research and practice

The most striking feature of the selected indicators is their low GRADE scores: the evidence base appears weak. This is in contrast to the reported evidence base for the quality of acute hospital-based paediatric care reported by Stang *et al* where 37% per cent of the selected indicators are based on moderate or high-quality evidence.^24^ To some extent this must reflect a lack of evidence about the optimal way of providing high-quality primary care to children; it also reflects the failure to select indicators from areas of care where the evidence is strongest (for example, vaccination). And perhaps it also reflects the nature of much of the existing evidence base with its focus on the absolute effectiveness of drugs and procedures rather than the optimal mechanism of their use (for example, when, to whom, and how much medicine is given).

Experience of the QOF in the UK has highlighted the importance of pilot-testing quality indicators before wide-scale national roll-out.^21^ The target population has to be carefully defined, precise coding developed to extract reliable information from computerised records, and the potential for gaming and the generation of unexpected adverse outcomes assessed. But it is important that care quality for children and adolescents is not ignored. Also, it is important that auditable quality indicators cover the full range of care. The indicator set that has been developed here reflects professional concerns and existing guidelines. In the UK it would be implementable through computerised audit of existing primary care records, whether or not it was used just as an audit tool or linked to QOF payment by results. So while the indicator set is not perfect, it is a start and improvement is most likely if it is taken up and used.
